# Decoding Local Field Potentials for Neural Interfaces

**DOI:** 10.1109/TNSRE.2016.2612001

**Published:** 2016-11-14

**Authors:** Andrew Jackson, Thomas M. Hall

**Affiliations:** Institute of Neuroscience, Newcastle UniversityNewcastleNE2 4HHU.K.

**Keywords:** Biofeedback, brain–machine interface (BMI), decoding, local field potentials (LFPs)

## Abstract

The stability and frequency content of local field potentials (LFPs) offer key advantages for long-term, low-power neural interfaces. However, interpreting LFPs may require new signal processing techniques which should be informed by a scientific understanding of how these recordings arise from the coordinated activity of underlying neuronal populations. We review current approaches to decoding LFPs for brain–machine interface (BMI) applications, and suggest several directions for future research. To facilitate an improved understanding of the relationship between LFPs and spike activity, we share a dataset of multielectrode recordings from monkey motor cortex, and describe two unsupervised analysis methods we have explored for extracting a low-dimensional feature space that is amenable to biomimetic decoding and biofeedback training.

## I. Introduction

Recent years have seen extraordinary progress in the development of brain–machine interfaces (BMIs) that use neuronal action potentials (spikes) recorded by implanted electrode arrays. Multichannel spikes from motor areas of the brain can provide control signals for computer interfaces, robotic prostheses, functional electrical stimulators and other assistive devices [1]. These BMI technologies, developed first in monkeys [2]–[5], are now being translated to human use [6]–[8] and hold considerable promise for improving the lives of paralyzed individuals. Moreover, neuroprostheses that use neural recordings to control electrical stimulation can reconnect parts of the nervous system that have been disconnected by injury [9], and concurrently drive neuroplastic changes that could help to rehabilitate function [10]–[12]. However, the clinical translation of spike-based BMIs and neuroprostheses faces two major challenges:

### A. Long-Term Stability

Most chronic electrode arrays are fabricated from materials that are mechanically incompatible with brain tissue. Micromotion of recording sites relative to neurons leads to changes in the shape of recorded action potentials, making consistent spike sorting a computational challenge. As the composition of spike recordings changes with time, so the performance of static decoders deteriorates. Therefore, most practical BMIs require some form of off- or on-line recalibration on a daily basis, even after electrodes have been implanted for many years. Recalibration can be time-consuming and technically/computationally challenging. Furthermore the number of neurons recorded by implants gradually reduces over months to years [13], [14] and thus the performance of decoders will decline even with daily recalibration. This deterioration is due to insulation degradation and mechanical breakage [14], [15], as well as a biological foreign-body response to injury—including cytokine release, reactive astrogliosis and microgliosis [16] that leads progressively to scarring [17] and neuronal death around electrodes [18]. New recording array designs have been proposed to improve long-term performance, for example by altering the biomechanical [19] and surface properties [20], [21] of electrodes, or by local delivery of immunosuppressants [22], [23]. However, obtaining long-term stable recordings of the same single neurons remains a considerable challenge at present.

### B. Sampling Frequency

Spike events are brief (<1 ms), and detecting or discriminating spike activity involves digitizing and transmitting signals at sampling rates of at least 10 kHz. Many current BMI implementations use percutaneous connectors and cables to convey signals to large, mains-powered electronics for processing. This presents a risk of infection and the general consensus in the community is that a move to subcutaneous implants, with wireless communication/power, will be required for widespread clinical uptake. However, the power consumption of wireless transmission increases with bandwidth and current devices for streaming spike data have battery lifetimes of a few hours. Note that while the frequency content of raw spike data extends up to the kilohertz range, the firing rates of individual neurons rarely exceed 100 spikes per second. Moreover, subsequent processing typically involves the estimation of firing rates by smoothing/filtering spike events over periods of approximately 100 ms, comparable to the time-scales of movement. Therefore the bandwidth required for relevant signal transmission can be reduced by migrating additional processing such as spike detection, sorting and firing rate estimation to implanted hardware [24]–[26]. However, this increases the power consumed by computational elements of the implanted circuitry. At present it is not clear whether wide-band transmission followed by external processing or a fully-implanted approach will be most suited for particular BMI applications. In either case, the power demands associated with high sampling frequencies are likely to remain a major impediment to subcutaneous implants.

## II. Local Field Potentials

Local field potentials (LFPs) could offer an attractive solution to both of these problems facing spike-based BMIs and neuroprostheses. The extracellular potentials recorded by electrodes in motor cortical areas typically comprise multiple components in distinct frequency bands which may contain movement-related information. Broad-band power at high frequencies (high gamma: 60–200 Hz) is generally positively correlated with neuronal firing rates [27], [28] and may reflect the summation of action potentials and/or synaptic currents associated with desynchronized, strongly active neuronal populations [29]. At intermediate frequencies, synaptic and intrinsic currents associated with neurons are synchronized to well-known sensorimotor rhythms (alpha: 7–13 Hz and beta: 15–30 Hz) that appear as narrow-band LFP oscillations. Finally, a low-frequency signal (lf-LFP: <5 Hz) has been termed the local motor potential (LMP).

Since the LFP reflects the summation of multiple sources in an extended volume around the recording site, it may be less sensitive to small movements of, or loss of cells near, the electrode tips [30]. BMIs using LFPs as control signals have been reported to be stable for many days to months [31]–[34]. Estimates of the region of tissue contributing to the LFP range from at least a few hundred micrometers around the recording site [35], [36] to over a centimeter [37]. However, modelling studies suggest that the spatial reach should be frequency-dependent [38], with high gamma signals arising from more local neural populations. If so, the high gamma signal may suffer similar instabilities as the spikes themselves. This is supported by an analysis of human intracortical recordings, in which a strong correlation was found between the performance of decoding based on high gamma power and multiunit spiking recorded on the same electrodes [39]. However, long-term recordings in monkeys suggest movement-related information can be present in the LFP signal from electrodes even in the absence of clear spike activity [33], [40], and a recent analysis of concluded that both the LMP and high gamma signals within the LFP are more stable than multiunit spiking [34].

Due to their frequency content, LFPs can be sampled, processed and/or transmitted at much lower rates than spike events. This is particularly true for the LMP, which varies on a time-scale comparable to movement kinematics. As a result, sampling rates of tens of Hertz rather than tens of kilohertz could in theory be used without violation of Nyquist’s theorem. This has a profound implication for the development of implantable interfaces: a reduction of sampling rates by three orders of magnitude could increase the battery lifetime of implanted devices from days to years.

Although the stability and power requirements for LFP recording offer considerable advantages for neuroprosthetics applications, the spatial averaging inherent in the signal poses challenges. Consistent with a frequency-dependent spatial reach to the LFP, low-frequency components in particular are highly correlated [40] and some studies have questioned whether such redundant signals could ever be as informative as spike recordings [40]–[42]. Direct comparisons between the information content of these signals have been attempted by a number of groups with conclusions ranging from LFPs performing somewhat worse [42]–[44], similar to [31], and even slightly better than spikes [45]. Stavisky et al. [33] reported that low-frequency LFPs were comparable to spikes for off-line decoding, although spikes performed better in closed-loop BMI experiments. The discrepancy in these various findings likely reflects differences in decoding algorithms, electrode geometries as well as experimental paradigms. However, in general these studies used electrode designs and decoders that have been optimized for spike recordings. Our view is that we will not be in a position to definitively assess the information content of LFPs until appropriate methods have been tailored to the peculiarities of the LFP signal. Optimizing an LFP-based neural interface may require the exploration of not only new signal processing methods, but also different decoding paradigms and electrode geometries. To approach this problem we will first review existing approaches that use LFPs for BMIs and, where relevant, consider lessons learned from spike-based interfaces, before suggesting several directions that we believe could be fruitful for future research.

### A. Biomimetic Decoding Strategies

At present, most BMIs use a “labelled” training set of recordings made during actual movements with known kinematics (e.g., speed/direction) or muscle activity. Decoders are typically trained using some form of supervised machine learning approach such as linear regression, generalized linear models, support vector machines or Bayes classification. The aim of a “biomimetic” decoder is thus to accurately estimate the observed behavior (output variables) based on some chosen biological control signals (input features). This approach has proved successful with firing rate inputs, and the same principle has been extended to other signal including intracortical LFP and surface electrocorticography (ECoG). Early studies compared the power in different frequency bands as input features [40], [45]–[51]. A consistent finding was that intermediate frequencies in the alpha and beta bands performed poorly in decoding studies, presumably because these sensorimotor rhythms are suppressed during movement. By contrast, information about movement could be retrieved from high (gamma band >50 Hz) frequencies, consistent with the theory that these reflect local spike firing rates. In addition, information was consistently reported at low-frequencies within the LFP. It is now clear that both kinematics [33], [40], [44], [46], [49] and electromyogram activity [51], [52] can be decoded from the LMP with considerable success, and this signal can out-perform the high-frequency bands [33]. Furthermore, models based on linear superposition of the LMP in the time domain generally outperform those based on power in the low-frequency band [33], [51], suggesting that the instantaneous phase of the low-frequency LFP can provide information additional to that contained within the amplitude signal alone.

For both spike- and LFP-based decoders, the biomimetic approach is problematic in practical applications with paralyzed patients since the training data must comprise imagined movements which may recruit different neural activity to that seen during closed-loop operation. To an extent this can be mitigated by closed-loop decoder adaptation (CLDA), wherein the decoder is adjusted based on activity patterns employed during on-line operation. CLDA has been applied successfully to both spike- [53], [54] and LFP-based decoders [55]. Nevertheless CLDA still requires “labelled” data (e.g., the intended direction of movement inferred from an instructed target) and can therefore only build decoders appropriate for a given training set (e.g., center-out arm movements from a central location). In general there is no a priori guarantee that any such decoder will generalize to behaviors/contexts outside of this set (e.g., movements starting in different regions of the workspace) [56]. Moreover, it is not obvious how this approach could be generalized to predict muscle activity (for control of functional electrical stimulation) or to higher cortical areas where the encoded parameters may be unknown and hence a “labelled” training data set may be impossible to obtain.

### B. Biofeedback Learning

An alternative to “biomimetic” decoding is the “biofeedback” approach, which exploits our capacity to learn new motor skills based on the sensory consequences of motor actions [56]. For example, we quickly adapt to visuomotor rotations, mirror reversals or abstract myoelectric interfaces that map muscles arbitrarily to new directions of movement [57]. Given appropriate feedback, monkeys and humans can learn to increase or decrease the firing rate of individual neurons in a variety of brain areas [58]–[61]. No “labelled” training data is required for biofeedback. Instead reward and/or error signals drive increased volitional modulation of the appropriate brain signals through operant conditioning. In principle, the biofeedback approach can be applied to any modality of neural recording and it has long been explored in relation to electroencephalogram (EEG) signals [62]. For example, with extended training subjects can learn to control a two- or three-dimensional cursor using desynchronization of sensorimotor rhythms originally associated with imagined movements of the hands and feet [63], while a similar biofeedback approach has been successful with high-gamma ECoG signals [64]. In addition it has been shown that gamma band LFP oscillations can be volitionally controlled in a biofeedback BMI paradigm [65].

Biomimetic decoding and biofeedback learning are not mutually exclusive, and it is likely that biofeedback-driven adaptation contributes to performance improvements during closed-loop operation of biomimetic decoders [2], [7]. Moreover biofeedback learning and CLDA can occur in parallel [66], ideally resulting in increased volitional modulation of input signals that are then appropriately mapped to desired outputs.

## III. Feature Extraction From LFPs

In supervised machine learning problems, particularly with high-dimensional, correlated inputs, the robustness and generalization of models can be improved by appropriate feature extraction/selection. If the input features to a decoder reflect consistent structure within neural signals, the impact of unstructured noise is reduced in the feature space compared to the full signal space. As a result, decoders based on a reduced set of principal components (PCs) of neural activity are more robust than those based on individual neural firing rates in the face of progressive neuron loss [67]. A related approach is to exploit consistent dynamical structure in neuronal recordings to reduce the impact of noise and improve robustness of decoders. Churchland et al. [68] described an underlying rotational structure in the firing rates of motor cortical neurons during arm movements, which could be visualized using the “jPCA” algorithm to project the high-dimensional data onto 2D planes that best captured the rotation. Incorporating knowledge of this dynamical structure into decoders improved performance in closed-loop BMI tasks [69].

Unlike biomimetic decoding, the biofeedback learning problem is one that must be solved by the brain rather than the system engineer. Biofeedback control signals can be chosen based on their stability over time, or other desirable characteristics, rather than simply whether they can predict movement parameters in a training dataset. Nevertheless the choice of which features to use as inputs, and how these should be mapped to outputs, is undoubtedly critical for the ease of learning and ultimate performance obtained. Even if any component of the input signals is, in principle, under volitional control (and this is not necessarily the case), learned strategies will likely be confined to a low-dimensional manifold, since feedback of success or errors will be insufficient to guide a full search of the high-dimensional control space. Thus efficient acquisition of accurate control will be facilitated if the biofeedback interface reflects the intrinsic structure of control signals. This principle has been successfully applied to body-machine interfaces, which can be operated by partially-paralyzed individuals using residual motion picked up by inertial sensors [70], [71]. The sensor data is projected onto the first two PCs calculated from a training dataset of free “dancing” movements. These PCs thus capture the sensor subspace that can easily be explored and utilized for control of computer cursors, wheelchairs or other assistive devices. Similarly, there is evidence that the neural adaptation involved in learning to control a spike-based BMI is constrained to a low-dimensional intrinsic manifold that can also be approximated by a small number of PCs [72]. Perturbations to decoders that require adaptation outside of this subspace are not learned as effectively as those for which neural solutions remain within the manifold. This suggests that biofeedback decoders that reflect the intrinsic low-dimensional structure of neural dynamics will be more successful than those that randomly map inputs to outputs.

These arguments suggest that for either biomimetic or biofeedback approaches, the appropriate choice of feature extraction and dimensionality reduction will simplify the learning process that must be solved by the decoder or the brain. This may be especially true for decoders based on multichannel LFP signals. Much, if not all, of the information in spike trains recorded from the motor cortex is conveyed by the firing rate of neurons. Therefore the first processing step in virtually all spike-based BMIs is to extract firing rates of individual neurons or multiunit activity. By contrast there is no such consensus about which are the appropriate features to extract from multichannel LFP as a first processing step.

As described above, LFPs contain components at many different frequency bands which likely reflect very different underlying neuronal processes. Although we do not discount the utility of high gamma signals for BMIs, we will here focus on low-frequency LFP signals (<5 Hz) since the LMP has consistently been found to be useful for decoding kinematics. Furthermore, the low sampling rate and larger spatial reach of this signal may be particularly advantageous for the key challenges of power consumption and stability of implanted devices. Within this frequency band, both the amplitude and phase content of the signals are likely to be important. Moreover, due to volume conduction and coordinated firing within the neuronal population, LFPs recorded on multielectrode arrays cannot be treated as independent channels of information. The challenge of interpreting multiple LFPs may be likened to the well-known “cocktail party problem” whereby each recording captures a mixture of multiple underlying sources. Appropriate features should thus reflect the correlation structure within the LFP signals. While it is possible to treat LFP decoding as a “black box” problem, it is valuable to acknowledge that LFP signals must arise in a lawful way from the anatomy and connectivity of underlying cortical circuits. We believe that an improved understanding of the nature of the LFP signal and how it relates to ongoing brain processes is vital to maximize its potential for neuroprostheses. Based on an ongoing program of research aimed at addressing this neuroscientific question, we suggest two possible strategies for extracting features from multichannel LFPs that may be suitable for low-power neural interfaces. Both approaches require a training dataset of neural recordings, but neither requires knowledge of the movements associated with that brain activity. As such these approaches are suitable for initial “unsupervised” feature extraction in a range of neuroprosthetic applications including (but not limited to) biomimetic and biofeedback paradigms.

### A. Decoding Neural Components From LFPs

One approach to LFP feature extraction is to find components within the multichannel signal that reflect concurrently-recorded spike activity from local neurons [73]. A single LFP signal can be modelled as a sum of the spike trains from multiple neurons (recorded on neighboring electrodes) convolved with suitable LFP waveforms (see also [74]). Formally, we use a system identification approach to fit the data as a multiple-input (spike trains) and single-output (LFP) linear time-invariant system with acausal impulse response functions. We use the term “spike-related slow potential” (SRSP) to describe the contribution of each neuron to the LFP (the impulse response function associated with each spike train). A computationally-efficient method for multiple-input single-output (MISO) system identification was provided by Perreault et al. [75] utilizing auto- and cross-correlation functions between inputs and outputs. The resultant SRSPs are conceptually similar to conventional spike-triggered averages which capture the cross-correlation between a single spike train and the LFP [74], [76]. The MISO approach additionally accounts for auto- and cross-correlation structure in the inputs, such that the contribution of correlated spikes in the recorded spike trains is removed from the SRSP attributed to each neuron (although the contribution of correlated but unrecorded neurons cannot be accounted for).

**Fig. 1.**
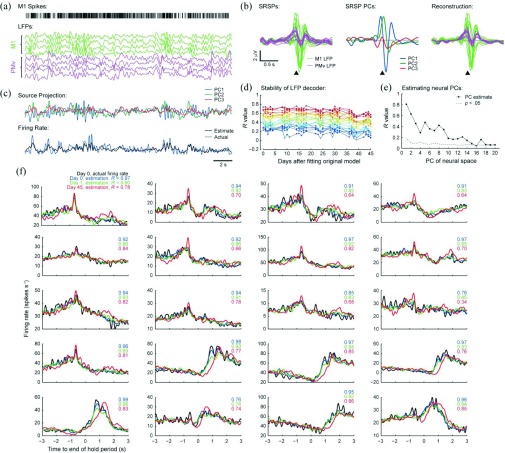
(a) Spikes from an example single neuron, and example low-frequency (<5 Hz) LFPs recorded from monkey primary (M1) and ventral premotor cortex (PMv). (b) Spike-related slow potentials (SRSPs) for the example neuron, showing the contribution of spikes from this neuron to a multiple-input, single-output model of each LFP channel (the model includes inputs from other recorded neurons that are not shown). The variation of SRSP across LFPs can be well-approximated by three principal components (PCs). (c) Source projections show the linear mixtures of LFP channels that best approximate the contribution of each SRSP PC to the LFP. Weiner deconvolution was then used to estimate neuronal firing rate. Plot shows performance on validation data not used to build the model. (d) Firing rate estimates for 20 neurons using a model built on day 0 were stable for 45 days. (e) Estimates of firing rates accurately capture the lowest PCs of the multichannel firing rate. (f) Trial-averaged modulation of the firing rate estimates of 20 neurons resemble the actual modulation of neurons on day 0 even after 45 days. Adapted from [73].

Importantly, recordings from monkey motor cortex [Fig. 1(a)] show that the SRSP associated with a given neuron varies substantially across different LFPs recorded on a multielectrode array [Fig. 1(b)]. In our data, this variation can generally be captured by linear mixtures of 3–4 sources which likely reflect different synaptic and/or intrinsic currents within the local cortical network. As a result there exists a low-dimensional projection of the multichannel LFP from which the firing rate of an individual neuron can be retrieved using Wiener deconvolution [Fig. 1(c)].

Once model parameters have been calculated, firing rates can be estimated in real-time using simple finite impulse response (FIR) filters applied to the LFP, requiring minimal computational resources whilst achieving surprisingly good performance. When tested on validation data not used to build the model, the instantaneous firing rate of single neurons could be estimated with Pearson’s R values from 0.2 to 0.7. Estimates based on a model built on day 0 remained surprisingly stable, although the correlation with actual firing rates deteriorated slightly over several weeks in which the neurons were recorded [Fig. 1(d)]. Note however that this decline could be due to instability in the LFP or the spike recordings (or both). To distinguish these, we examined the relationship between actual/estimated firing rates and movements made during an isometric wrist torque task. Movement-aligned average firing rates could be reconstructed with R values greater than 0.9 [Fig. 1(f)], and on subsequent days the estimated firing rates (using the model built on day 0) out-performed the actual firing rates at reproducing the original pattern of modulation [73]. Indeed, consistent task-related firing rate profiles could be retrieved from LFPs for the duration of the recording period (up to 116 days in one subject) by which point many of the original neurons had been lost, suggesting that LFP signals (at least in the low-frequency range) can be informative even in the absence of spiking activity. The estimates were of sufficient quality to enable biofeedback control with monkeys readily able to increase or decrease the firing rate estimates to reach high/low targets. This was achieved by appropriate and selective modulation of the actual neuronal firing rates [73].

Of particular pertinence to the present review, the lowest PCs (i.e., those capturing the most variance) within the firing rates of a neural population can be estimated with even higher precision than individual neurons [Fig. 1(e)]. Therefore the LFP seems particularly suited for decoding the same population components that have previously been identified as being an appropriate feature space for both biomimetic and biofeedback BMIs. We suggest that early in the life of an electrode implant, a decoder could be built that maps multichannel LFP to the firing rates (or firing rate PCs) of neurons recorded with high fidelity. The output of this decoder could augment or replace conventional firing rate estimates using spike activity, and performance should outlast that obtained from spikes alone as recordings deteriorate.

### B. Areal Velocity Decoding

The previous approach requires an initial training dataset containing spike recordings, and it can therefore only estimate the activity of those neurons recorded concurrently on the electrode array. But if information about the firing of individual neurons is contained within the LFP, could it be possible to extract these features without any prior information about spiking activity? To explore this question we considered how the SRSPs from multiple neurons are combined in the LMP during an isometric wrist torque-tracking task [77].

**Fig. 2.**
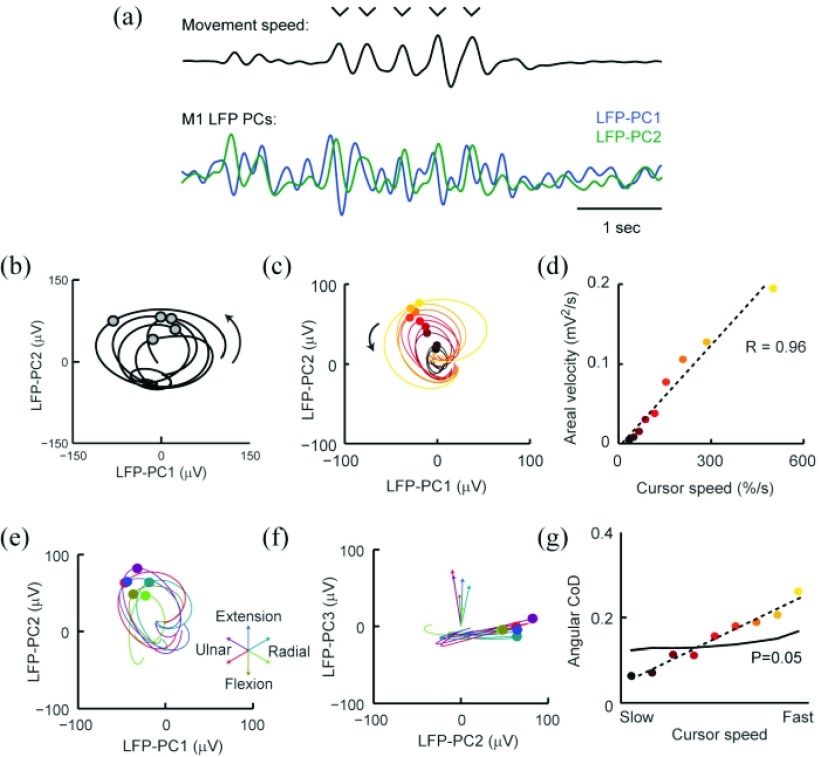
(a) Cursor movements during an isometric wrist torque-tracking task show rhythmical speed fluctuations associated with submovements (indicated by tick marks). The first two PCs of M1 LFPs exhibit orthogonal components of an oscillatory cycle phase-locked to submovements. (b) Trajectory of M1 LFP PCs reveals consistent rotational structure. (c) Submovement-triggered average LFP cycles binned according to submovement speed. (d) Areal velocity of submovement-triggered average trajectories is linearly proportional to submovement speed. (e) Submovement-triggered average trajectories for LFP PC1 versus LFP PC2 binned according to submovement direction. (f) Submovement-triggered average trajectories for LFP PC2 versus LFP PC3 binned according to submovement direction. In this projection, it can be seen that the LFP trajectory for different submovement directions rotates around slightly different axes. (g) Submovement direction decoded from the 3D areal velocity of LFP PC trajectories. Decoding accuracy is measured with an angular coefficient of determination (CoD) and compared against the 95th percentile performance on shuffled data. Adapted from [77].

Many upper-limb movements comprise multiple submovements which occur at 1–4 times per second [Fig. 2(a)] and are associated with phasic neural activity in primary and premotor cortices. The combined SRSPs associated with this activity can be observed in the LFP as an oscillation at the same frequency. Therefore, increased power in the low-frequency LFP is observed during movement, but this may be hard to distinguish from other sources of physiological signals and recording artefacts containing low-frequency components. However, since the SRSP from local neurons comprises several sources with different spatio-temporal profiles, the contribution of these neurons appears with a different phase in different LFP channels. Therefore principal component analysis (PCA) yields two orthogonal components of the underlying movement-related oscillation (effectively a sine and cosine with a frequency of 1–4 Hz), which can be visualized by plotting the trajectory of the LFP in the PC plane [Fig. 2(b)]. Submovements during an isometric torque-tracking task are associated with a single cycle of the oscillation rotating in a consistent direction [Fig. 2(c)], reminiscent of the cycles previously reported for the firing rates of neuron populations during reaching [68].

A simple and convenient metric to quantify the amplitude of LFP cycles is this is the areal velocity, defined as the area swept out per unit time by the high-dimensional LFP, $\boldsymbol {l}$, in the plane defined by the first two PCs, $\boldsymbol {p}_{1}$ and $\boldsymbol {p}_{2}$. This can be calculated from the cross-product of the LFP projection with its time-derivative

\begin{align*} {AV}_{\boldsymbol {p}_{1},\boldsymbol {p}_{2}}&= \frac {1}{2}\left [{{ {\begin{array}{*{20}c} \boldsymbol {p}_{1}.\boldsymbol {l}\\ \boldsymbol {p}_{2}.\boldsymbol {l}\\ \end{array}} }}\right ]\times \left [{{ {\begin{array}{*{20}c} \boldsymbol {p}_{1}.\dot {\boldsymbol {l}}\\ \boldsymbol {p}_{2}.\dot {\boldsymbol {l}}\\ \end{array}} }}\right ]\notag \\ &= \frac {1}{2}\left ({{\left ({{\boldsymbol {p}_{1}.\boldsymbol {l} }}\right )\left ({{\boldsymbol {p}_{2}.\dot {\boldsymbol {l}} }}\right )\mathbf {-}\left ({{\boldsymbol {p}_{2}.\boldsymbol {l} }}\right )\left ({{\boldsymbol {p}_{1}.\dot {\boldsymbol {l}} }}\right ) }}\right ). \end{align*}

**Fig. 3.**
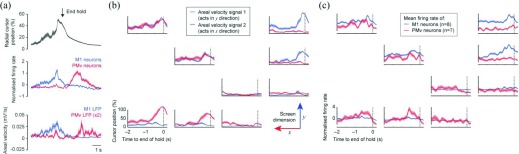
(a) *Top:* Radial cursor position aligned to the end of successful hold periods for peripheral targets in an isometric wrist torque-tracking task, averaged across 40 trials (shading shows standard error of the mean). *Middle:* Average normalized (to zero mean and unity standard deviation) firing rate for eight M1 neurons and six PMv neurons aligned to the end of the hold. *Bottom:* Average areal velocity in the first PC plane of M1 and PMv LFPs aligned to the end of the hold. The profile of areal velocity during task performance mirrors the dissociation seen in neural activity across areas. Adapted from [77] (b) Cursor position in a two-dimensional BMI task controlled by two areal velocity signals. The figure lay-out represents the locations of nine targets in this two-dimensional task. The origin is at the bottom right corner, and each sub-panel is drawn at the approximate screen location of a target. The “jPCA” technique (see text) was used to find the two planes from a full space of 23 LFPs which best captured rotational structure. The areal velocity signal from planes 1 (blue) and 2 (red) controlled the y and x dimensions of the task, respectively. Each sub-panel shows average areal velocity signals aligned to the end of the successful hold period (39 trials per target). The monkey was able to generate independent (targets along axes) and simultaneous (targets on diagonal) areal velocity in each plane. (c) Average normalized (zero mean and unity variance) firing rates of eight M1 neurons (blue) and seven PMv neurons (red) during the same task. Areal velocity in plane 1 is associated with increased firing rates in M1, while areal velocity in plane 2 is associated with increased firing rates in PMv.

We find that the areal velocity of M1 LFP cycles associated with submovements is proportional to submovement speed across a wide range [Fig. 2(d)]. Moreover, the trial-averaged profile of areal velocity in primary motor cortex (M1) and ventral premotor cortex (PMv) matches the different time-course of neural activity in each area during our task [Fig. 3(a)].

These observations led us to wonder whether the areal velocity of LFP cycles would be amenable to biofeedback control. To begin answering this question, we have applied the “jPCA” algorithm [68] to multichannel LFP data recorded from M1 and PMv, to extract two planar projections that maximize rotational structure. For the example session shown in [Fig. 3 (b),(c)], the first plane corresponded mostly to M1 LFPs, while the second mainly captured PMv LFPs (i.e., the algorithm effectively separated the dynamics associated with each cortical area). We calculated in real-time the areal velocity in each plane and used these signals to control the 2D position of a biofeedback cursor. We found that monkeys could readily produce rotation in one plane only or both together to reach a variety of targets [Fig. 3(b)]. This behavior was associated with distinct modulation of the underlying neuronal firing rates in M1 and PMv [Fig. 3(c)] suggesting that each area can generate independent low-frequency neural dynamics that are reflected in the multichannel LFP.

## IV. Future Research Directions

We hope that the examples presented above demonstrate the great potential of applying new signal processing techniques to LFP recordings. We have described an approach to decoding neural firing rates from LFPs using linear FIR filters, but more sophisticated methods may improve performance, for example using Kalman filters that incorporate a model of intrinsic neural dynamics [69]. To date, we have used only the low-frequency LFP, but additional information about spiking may also be obtained from higher frequency bands [76]. Furthermore, in decoding firing rates from LFPs, we have first used linear methods for simplicity, but it is possible that non-linear transformations of the LFP may yield more informative features. The areal velocity swept out in the PC plane is one such non-linear transformation (since its calculation involves the multiplication of two components derived from the LFP), and we speculate that this approach could be extended to extract further information from LFP signals. For example, in the space defined by the first three PCs, we find that the LFP trajectories associated with submovements in different directions trace rotational cycles around slightly different axes. Effectively, the first two PCs reflect LFP dynamics that are consistent across all submovements [Fig. 2(e)], while the third component captures subtle variations in the SRSPs arising from directionally-tuned neuronal populations [Fig. 2(f)]. In three dimensions, the areal velocity cross-product yields a vector with both magnitude and direction, allowing decoding of both submovement speed (from the vector magnitude) and submovement direction [from the vector direction; Fig. 2(g)]. More generally, consider the projection of the high-dimensional LFP vector onto an arbitrary plane spanned by orthogonal vectors $\boldsymbol {u}$ and $\boldsymbol {v}$. The area velocity in this plane is a linear sum of pairwise areal velocity terms

\begin{align*} {AV}_{\boldsymbol {u},\boldsymbol {v}}&= \frac {1}{2}\left [{{ {\begin{array}{*{20}c} \boldsymbol {u}.\boldsymbol {l}\\ \boldsymbol {v}.\boldsymbol {l}\\ \end{array}} }}\right ]\times \left [{{ {\begin{array}{*{20}c} \boldsymbol {u}.\dot {\boldsymbol {l}}\\ \boldsymbol {v}.\dot {\boldsymbol {l}}\notag \\ \end{array}} }}\right ]\\ &= \sum \limits _{i<j} \left ({{u_{i}v_{j}-u_{j}v_{i} }}\right ) {AV}_{i,j} \end{align*}

where

\begin{equation*} {AV}_{i,j}=\frac {1}{2}\left ({{l_{i}\dot {l}_{j}-l_{j}\dot {l}_{i} }}\right ). \end{equation*}

**Fig. 4.**
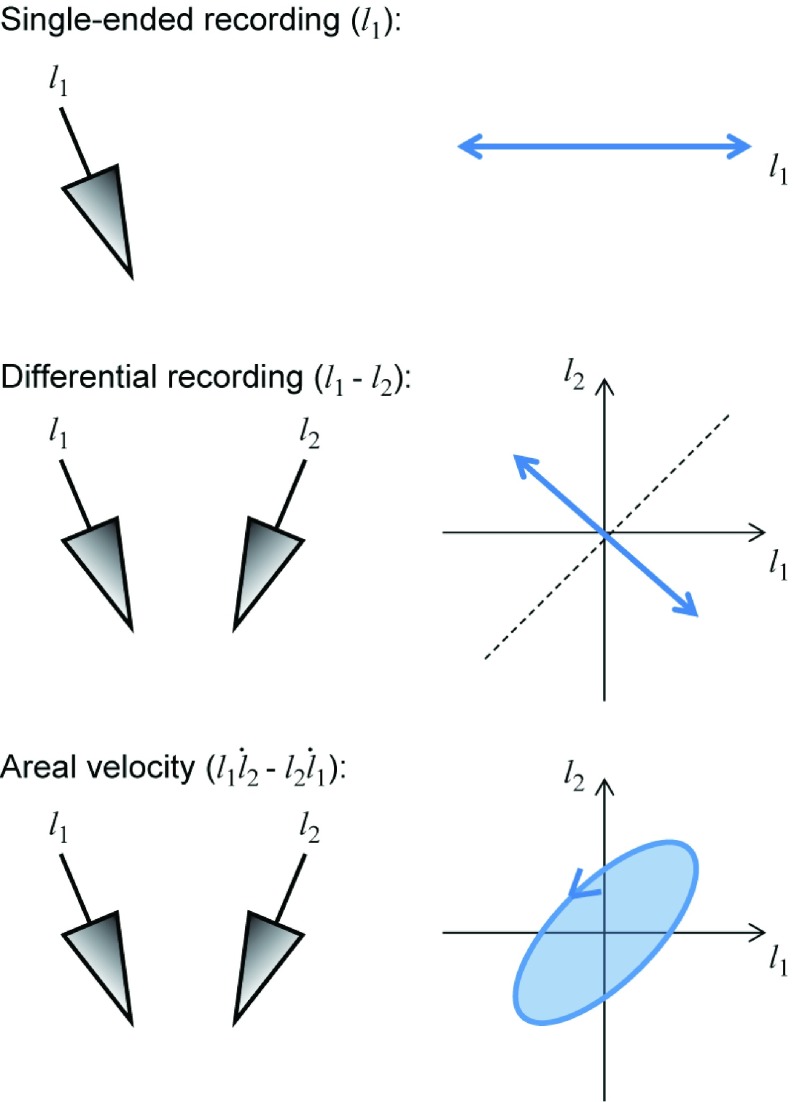
Comparison of different recording methods. Differential recording rejects signal components that are common to two LFPs. Areal velocity calculation rejects any in-phase oscillatory components in the two LFPs.

Note that these pairwise areal velocity signals, ${AV}_{i,j}$, are conceptually similar to differential recordings (Fig. 4). A differential recording rejects common signal in both channels, and is therefore sensitive to physiological sources which are recorded with different amplitudes within the multichannel LFP. By contrast, the areal velocity signal rejects all in-phase correlated components (regardless of their amplitude) and is therefore effective at selecting only those physiological sources that produce consistent phase differences within the multichannel LFP. Note also that the mean power of a differential signal is always non-zero due to inevitable sources of background noise. However, in the absence of SRSPs, the areal velocity will on average be zero since there is no consistent phase difference in the noise on different channels. As a result areal velocity reflects local neuronal activity but is relatively insensitive to both distant sources and changes background noise levels. We therefore suggest that pairwise areal velocity signals may provide useful features for both biomimetic and biofeedback decoding approaches, especially since their calculation is relatively simple to implement in low-power hardware.

The use of non-linear transformations such as areal velocity can expand the dimensionality of the feature space (since N LFP channels yields $\raise 0.7ex\hbox {1} \!\mathord { /{ {\vphantom {1 2}}} }\!\lower 0.7ex\hbox {2}N(N-1)$ LFP pairs). Moreover, multiple areal velocity signals can be obtained separately for each frequency within the LFP by adding preliminary band-pass filtering. (Alternatively, a related transformation can be applied in the frequency domain by calculating the complex part of the pairwise cross-spectra). However, it is likely that activity will be constrained to only a small portion of this high-dimensional space. An important area of future research will therefore be to determine which and how many combinations of these areal velocity features are under volitional control.

A final consideration is the geometry of electrode array used to record field potential signals. Most LFP decoding studies use arrays in which all electrodes are at the same depth (for example Blackrock “Utah” arrays), which makes sense if the primary aim of experiments is to maximize spike recordings from neurons in a particular cortical layer, but may not be optimal for extracting information from the LFP. ECoG signals obtained from the brain surface have the advantage of being less invasive than penetrating electrodes and show good signal stability [78]. Indeed, low-frequency components are even present in the non-invasive EEG signals and can be used for kinematic decoding [79]–[81]. However, the SRSP varies across nearby LFP recordings and changes polarity as electrodes are advanced through the cortex [73]. This local structure may explain why LFP decoding generally out-performs ECoG and EEG signals [40], [48], [82] and suggests that the information content of LFPs could be improved further by placing electrodes at multiple depths to optimally capture the distinct SRSP components and reduce the redundancy of recordings. We believe the time has come to stop treating LFPs as a secondary signal recorded in addition to spikes (often analyzed only as an after-thought if spike recordings are poor), and instead design recording arrays specifically for maximizing the information content in the LFPs. We suggest therefore that future research should examine in detail the spatial distribution of the SRSP to establish systematically the optimal depths, size and spacing of electrodes for LFP decoding.

To stimulate research into new LFP decoding methods, we are making available a dataset arising from the experiments described above. The dataset consists of 31 short sessions of isometric torque-tracking task and includes spikes from 20 neurons (M1: 13, PMv: 7) and 22 LFPs (M1: 11, PMv: 11) collected over 46 days. These data could be used to test alternative methods of decoding either firing rates or kinematics from LFP signals, and compare their relative stability over time. We are willing to share further datasets from multiple subjects and advise interested researchers to contact the corresponding author (AJ).

## V. Conclusion

Interpreting and decoding LFP signals presents a number of unique challenges, and may require a different toolbox to spike-based BMIs and neuroprostheses. An improved understanding of how the LFP arises from coordinated activity within cortical networks will help determine which LFP features (e.g., amplitude/phase/frequency/correlation) reflect underlying neuronal activity and are amenable to biomimetic and biofeedback decoding strategies. In particular, we suggest features that capture the spatio-temporal structure of spike-related slow potentials whilst minimizing the impact of unstructured noise will be effective at maximizing the information that can be obtained from multichannel LFP.
